# GPEdit: the genetic and pharmacogenomic landscape of A-to-I RNA editing in cancers

**DOI:** 10.1093/nar/gkab810

**Published:** 2021-09-17

**Authors:** Hang Ruan, Qiang Li, Yuan Liu, Yaoming Liu, Charles Lussier, Lixia Diao, Leng Han

**Affiliations:** Department of Biochemistry and Molecular Biology, McGovern Medical School at The University of Texas Health Science Center at Houston, Houston, TX 77030, USA; Center for Epigenetics and Disease Prevention, Institute of Biosciences and Technology, Texas A&M University, Houston, TX 77030, USA; Center for Epigenetics and Disease Prevention, Institute of Biosciences and Technology, Texas A&M University, Houston, TX 77030, USA; Department of Biochemistry and Molecular Biology, McGovern Medical School at The University of Texas Health Science Center at Houston, Houston, TX 77030, USA; Center for Epigenetics and Disease Prevention, Institute of Biosciences and Technology, Texas A&M University, Houston, TX 77030, USA; Department of Computer Science and Statistics, Rice University, Houston, TX 77030, USA; Department of Bioinformatics and Computational Biology, The University of Texas MD Anderson Cancer Center, Houston, TX 77030, USA; Department of Biochemistry and Molecular Biology, McGovern Medical School at The University of Texas Health Science Center at Houston, Houston, TX 77030, USA; Center for Epigenetics and Disease Prevention, Institute of Biosciences and Technology, Texas A&M University, Houston, TX 77030, USA; Department of Translational Medical Sciences, College of Medicine, Texas A&M University, Houston, TX 77030, USA

## Abstract

Altered A-to-I RNA editing has been widely observed in many human cancers and some editing sites are associated with drug sensitivity, implicating its therapeutic potential. Increasing evidence has demonstrated that a quantitative trait loci mapping approach is effective to understanding the genetic basis of RNA editing. We systematically performed RNA editing quantitative trait loci (edQTL) analysis in 33 human cancer types for >10 000 cancer samples and identified 320 029 edQTLs. We also identified 1688 ed-QTLs associated with patient overall survival and 4672 ed-QTLs associated with GWAS risk loci. Furthermore, we demonstrated the associations between RNA editing and >1000 anti-cancer drug response with ∼3.5 million significant associations. We developed GPEdit (https://hanlab.uth.edu/GPEdit/) to facilitate a global map of the genetic and pharmacogenomic landscape of RNA editing. GPEdit is a user-friendly and comprehensive database that provides an opportunity for a better understanding of the genetic impact and the effects on drug response of RNA editing in cancers.

## INTRODUCTION

RNA editing is a unique type of post-transcriptional modification that alters specific nucleotide sequences originated from one organism's genome. The major form of RNA editing in metazoans is Adenosine to Inosine (A-to-I), a process that is catalyzed by the adenosine deaminase acting on RNA (ADAR) enzymes (1,2). RNA editing, for its potential effects on transcript functions, has been associated with human diseases such as neurological disorders (3) and carcinoma in multiple tissue types (4,5). Previously, the pan-cancer A-to-I editome has been comprehensively profiled using RNA-seq data (6), and the genome-wide altered A-to-I RNA editing patterns are observed across multiple cancer types. Those RNA editing events identified in tumors are contributing to transcript complexity, or could further increase diversity at proteomic levels, that eventually affect functions of cancer cells (7).

Previous works demonstrated that a quantitative trait loci (QTL) mapping approach is effective to understand the genetic basis of multiple molecular features in human cancers, e.g. gene expression, methylation, and alternative splicing (8–10). Several studies have been prioritizing the significance of RNA editing QTL (edQTL) in understanding relations between genetic variation and RNA editing functions (11–14). In these studies, the *cis*-regulatory mechanism of RNA editing patterns has been examined in animal models or normal human tissues. Genetic variants such as Single Nucleotide Polymorphism (SNP) that potentially affect RNA secondary structure are partially accounted for by the altered editing frequency. In human cancers, we have shown that RNA editing events are closely linked to clinical information such as patient overall survival (6). More importantly, some non-synonymous editing sites are associated with drug sensitivity, implicating a therapeutic role of RNA editing as potential targets (6). In other human diseases, RNA editing has been emerging as a biomarker in predicting treatment outcomes such as adverse drug reactions (15).

The significant impact of genetic variants on different noncoding RNAs (16,17) and post-transcriptional regulation are recognized recently (18). Furthermore, we previously demonstrated that RNA editing may impact on drug response (6), but the associations between drug response and RNA editing across large number of cancer samples are not investigated. Despite the importance of interpretation of the genetic and pharmacological landscape of RNA editing in human cancers, there are no data resources that provide either edQTL information or drug sensitivity relations of RNA editing on a large scale. To bridge this gap, we implemented a computational pipeline to systematically identify edQTLs in 33 cancer types incorporating ∼10 000 tumor samples from The Cancer Genome Atlas (TCGA). By adopting an established approach (19), we also imputed the drug response of TCGA patients from ∼1000 compounds in Genomics of Drug Sensitivity in Cancer (GDSC) (20) and Cancer Therapeutics Response Portal (CTRP) (21) and investigated their associations with RNA editing. The data were deposited into our newly developed database GPEdit (genetic, pharmacogenomic landscape of A-to-I RNA editing in cancers, https://hanlab.uth.edu/GPEdit/).

## DATA COLLECTION AND PROCESSING

### TCGA genotype data pre-processing

**Figure 1.**
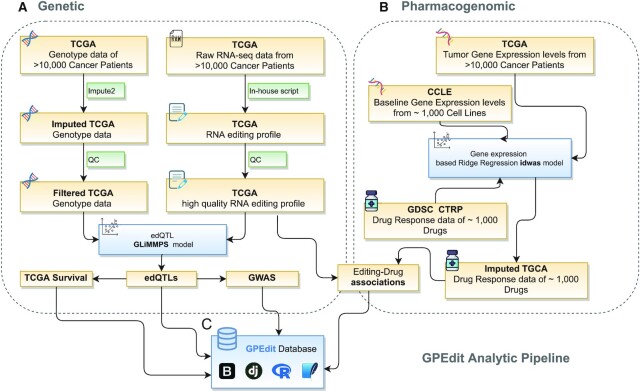
Analytic pipeline for data processing in GPEdit. (**A**) The pipeline to identify edQTLs, including processing and quality control of genotype data and RNA editing profiles, applying GLiMMPS model to identify edQTLs and crossmatch with survival information and GWAS catalogs. (**B**) The pipeline to identify drug response associated RNA editing events, including patients’ drug response imputation and large-scale correlation tests. (**C**) GPEdit database infrastructure.

TCGA level 2 genotype data from Affymetrix SNP Array 6.0 were downloaded from the Genomic Data Commons data portal (GDC; https://gdc.cancer.gov) (Figure 1A). As described in our previous publications (9,22), autosomal variants imputation helps to increase power for QTL discovery. We used IMPUTE2 (22) along with 1000 genome Phase 3 as a reference panel to perform the two steps of pre-phasing and autosomal variants imputation. Following quality control criteria were considered to exclude SNPs after imputation: (i) imputation score INFO < 0.4; (ii) minor allele frequency (MAF) < 0.05; (iii) SNP missing rate ≥0.05 for best-guessed genotypes with posterior probability ≥0.9 and (iv) Hardy–Weinberg equilibrium *P*-value < 1 × 10^–6^.

### Profiling of pan-cancer A-to-I RNA editing

We have previously developed a computational pipeline to characterize A-to-I RNA editing profiles in 17 TCGA cancer types following a RADAR reference panel (23) of ∼1.4 million A-to-I RNA editing sites (6). Here, we expanded our data collection to 33 cancer types following an updated reference panel of ∼4.5 million sites (24). We downloaded RNA-seq BAM files of 10 179 patient tumor samples from the GDC data portal. For sequence quality control, we only considered A-to-I RNA editing sites with at least 10 high-quality reads (base quality score ≥ 30) covered with at least two high-quality reads supporting the editing events (25). We further selected editing sites with editing frequency (edited reads / covered reads) significantly greater than 0.1% (binomial test with false discover rate controlled at 5%) (25) for the following edQTL analysis. We also excluded editing sites overlapping with variates annotated in dbSNP (version 138) and TCGA MC3 somatic mutations (26). To ensure sufficient editing frequency variations among cancer patients, a filter of a minimum 10% difference between the editing frequency of the 90% quantile and the 10% quantile across all tumor samples was implemented (14).

### Identification of edQTLs

To emphasize the cis-regulatory role of detected edQTLs, only SNPs within 200 kb of editing sites were included in our analysis (14). To test the associations between SNPs and A-to-I RNA editing frequencies, we adopted a generalized linear mixed model GLiMMPS (27), which was applied in edQTL analysis (14). For each editing site, the edQTL was defined as the closest SNP with the most significant GLiMMPS *P*-value. For edQTLs identified in each cancer type, false discover rate (FDR) was controlled as <0.01.

### Identification of edQTLs associated with survival and GWAS risk loci

EdQTLs were further examined against patients' overall survival time. For each edQTL, tumor samples were classified into three groups by their genotypes and log-rank tests were performed on them to examine the significance of the overall survival time difference among groups. Within each cancer type, an edQTL with FDR <0.1 was defined as survival associated edQTL. Kaplan-Meier (KM) curves were used to visualize the differences between groups.

Genome-wide association studies (GWASs) have been contributing to understanding relations between genetic risk loci and complex diseases (28). Here, we downloaded available risk tag SNPs from the NHGRI-EBI GWAS catalog (http://www.ebi.ac.uk/gwas/, access on September 2020) (29). GWAS linkage disequilibrium (LD) regions of these risk tags SNPs were obtained from the SNAP database (https://data.broadinstitute.org/mpg/snpsnap) (30). The European (EUR) population in the 1000G Phase 3 dataset was selected with LD cut-off *R*^2^ over 0.5. EdQTLs overlapped with GWAS tag SNPs were defined as GWAS associated edQTLs.

### Identification of drug response associated editing events

Recent studies demonstrated the significance of evaluating the drug response in patient samples (31,32). In this study, we adopted a previously established approach to impute the drug response of TCGA patient samples (idwas, https://osf.io/yatu3/) (19) and expanded to ∼1000 anti-cancer drugs available in other drug response data resources such as GDSC2 and CTRP (20,21). For each cancer type, we used the ‘rcorr’ function in the ‘Hmisc’ package to perform a large-scale Spearman Correlation test between RNA editing and drug response. We set a minimum of 50 samples of paired RNA editing frequency and imputed drug response. Editing-drug pairs with absolute Spearman Correlation over 0.3 and FDR <0.01 were considered as a significant association.

## DATABASE CONTENT AND USAGE

### Data summary

In the GPEdit data portal, we performed a comprehensive edQTL analysis across 33 TCGA cancer types to understand the genetic basis of the cis-regulatory mechanism in human cancers (Table 1). The total number of edQTLs detected in 33 cancer types was 320 029, of which around 10% edQTLs (31,842) were detected in more than one cancer type. The numbers of edQTLs identified in each cancer type were ranging 776 in Adrenocortical Carcinoma (ACC) to 41 832 in Stomach Adenocarcinoma (STAD). The power of QTL detection increases with cohort samples size (*R*s = 0.73, *P* = 1.19 × 10^–6^), which is consistent with our previous pan-cancer QTL studies (10,16). The total number of survival-associated edQTLs is 1,688, of which the highest is 243 in Esophageal Carcinoma (ESCA) and the lowest is zero in Glioblastoma multiforme (GBM) (Table 1). A total of 4672 GWAS-associated edQTLs were found, of which the highest is 607 in STAD and the lowest is 14 in Uveal Melanoma (UVM). We identified a total of 3 481 011 significant associations between drug response and editing events in 31 cancer types with sample size over 50. The number of significant pairs of associations ranged from nine in ACC to 658 619 in Thymoma (THYM) (Table 1).

**Table 1.** Data Summary of edQTLs for each cancer type in GPEdit

Cancer type	#Sample	#Editing events	#edQTLs	#Survival associated edQTLs	#GWAS associated edQTLs	#Editing drug pairs
ACC	79	5906	776	4	22	9
BLCA	408	22 535	7800	69	133	187 275
BRCA	1092	52 158	15 668	20	235	228 675
CESC	304	19 290	7046	49	99	47 386
CHOL	37	9 383	2075	8	36	0
COAD	456	20 355	6910	4	97	78 557
DLBC	48	6 931	1569	16	18	0
ESCA	164	95 264	39 244	243	575	42 208
GBM	149	27 316	7794	0	113	72 277
HNSC	501	16 371	6098	24	112	85 168
KICH	66	12 841	3 238	8	42	60
KIRC	530	43 326	17 793	37	267	192 376
KIRP	289	26 742	11 499	145	144	73 399
LAML	151	46 633	9815	30	142	1512
LGG	455	25 314	9042	44	123	339 182
LIHC	371	19 884	7495	68	101	75 823
LUAD	515	32 510	13 504	40	199	38 679
LUSC	501	33 142	12 936	47	204	25 923
MESO	86	10 573	2747	20	44	55
OV	389	69 877	18 314	16	263	30 375
PAAD	177	16 505	5974	26	100	30 162
PCPG	179	15 267	5182	86	67	21 983
PRAD	496	25 321	10 643	109	139	148 336
READ	167	11 630	3842	11	60	340
SARC	259	19 277	6794	62	86	40 870
SKCM	468	22 969	6519	71	94	98 009
STAD	380	100 755	41 832	161	607	37 285
TGCT	150	16 689	5 372	133	67	511 418
THCA	502	26 644	10 304	95	152	395 504
THYM	119	16 027	5251	12	80	658 619
UCEC	555	28 222	12 103	21	161	4794
UCS	56	11 231	3339	1	76	81
UVM	80	7239	1511	8	14	14 671

### Web design and interface

GPEdit data portal was constructed based on Django framework with Bootstrap as front-end web interface and SQLite as a back-end database tool (Figure 1C) (33). Various JavaScript libraries such as ‘DataTable’ were used in the web interface. R with ‘ggplot2’ package was used to produce figures that were deposited in GPEdit. GPEdit is freely available at (https://hanlab.uth.edu/GPEdit).

**Figure 2.**
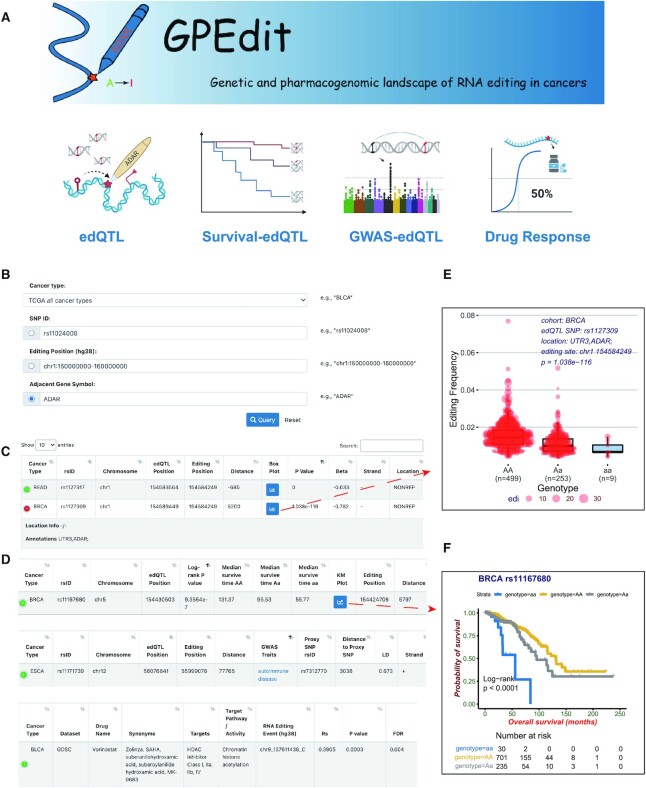
Overview of the GPEdit database. (**A**) Four functional modules are provided in GPEdit. (**B**) Query web interface in the ‘edQTL’ module. (**C**) An example of query return table of the ‘edQTL’ module. (**D**) Examples of query return tables from the ‘Survival-edQTL’ module (upper panel), ‘GWAS-edQTL’ module (middle panel), and ‘Drug Response’ module (lower panel). (**E**) An example of a box plot visualizing one edQTL. (**F**) An example of Kaplan-Meier plot visualizing one survival associated edQTL.

There are four function modules available (Figure 2A) in GPEdit for data query: (i) ‘edQTL’ for querying identified edQTLs; (ii) ‘Survival-edQTL’ for querying edQTLs that have significant associations with patients’ survival; (iii) ‘GWAS-edQTL’ for querying edQTLs that may link with SNPs that annotated in GWAS studies and (iv) ‘Drug Response’ for querying significant drug associated RNA editing events. The GPEdit also provides query function by cancer types on its home page.

Within each query function module, queries be made using a set of user-defined criteria such as SNP id (e.g. rs11024008), a specific genomic location (e.g. chr1:150000000-160000000), or the gene symbol (e.g., ADAR) adjacent to RNA editing sites (Figure 2B). For example, in the ‘edQTL’ module, when a user chose the query option by ‘Adjacent Gene Symbol’ and type in ‘ADAR’. The text box would automatically match and autofill with available gene symbols for convenience. After clicking the ‘Query’ button, a table with query returns would appear, and results matched with ‘ADAR’ would be listed in the table (Figure 2C). For example, the table shows two edQTLs that are associated with the same RNA editing sites located at the 3’UTR region of the gene ADAR.

For other modules, making a query is similar to the ‘edQTL’ module, while the query options could be different. For example, the ‘GWAS-edQTL’ module has an additional ‘Linkage disequilibrium’ cutoff choosing option. The query returns are also different among modules (Figure 2D). Some modules provide a data visualization option in the query returns table. For instance, the ‘edQTL’ module provides a box plot to visualize edQTLs’ impact on RNA editing frequencies among cancer samples (Figure 2E) and the ‘Survival-edQTL’ provides a Kaplan-Meier plot (KM plot) to visualize edQTLs’ association with patient's survival (Figure 2F). The box plot in Figure 2E shows an edQTL rs1127309 is significantly associated with RNA editing frequencies of an editing site located at the 3’UTR region of the gene ADAR. The KM plot in Figure 2F shows that an edQTL rs11167680 is significantly associated with patients' survival using the Log-rank test (*P* < 0.0001), and the risk table is listed below the KM plot.

Additionally, all the query return tables are searchable and can be downloaded in ‘Microsoft Excel’ compatible format. All the visualization plots can be saved in ‘png’ format as well as in ‘pdf’ format. A detailed tutorial of GPEdit can be found on the ‘Document’ page.

## SUMMARY AND FUTURE DIRECTIONS

We systematically investigated the genetic and pharmacogenomic basis of A-to-I RNA editing events in 33 human cancers. We constructed a user-friendly database, GPEdit, for users to query, browse and download edQTLs. Huge amounts of vector diagrams of edQTL box plots and KM plots are provided. GPEdit could serve as an important resource for human cancer genetics and provide opportunities to bridge the knowledge gap from variants in sequence to RNA editing. In addition, GPEdit provides the associations between RNA editing and >1000 anti-cancer drugs thus contributing to understanding the functional effects of RNA editing on drug response. Cancer Genomics is an explosively growing field in recent years (33–37), with the great effort from several large-scale consortiums, including TCGA, International Cancer Genome Consortium (38), as well as many other studies with a significant amount of data. We will periodically survey newly released cancer data resources with a considerable number of samples with matching genotype data, expression data, and drug response data. We will update GPEdit accordingly and maintain it as a useful resource for the research community.

## DATA AVAILABILITY

GPEdit is a data resource portal that is freely available at (https://hanlab.uth.edu/GPEdit). Analytic codes for RNA editing calling and edQTL detection are available at GitHub site (https://github.com/hr1912/GPEdit).
